# Computerized Hammer Sounding Interpretation for Concrete Assessment with Online Machine Learning

**DOI:** 10.3390/s18030833

**Published:** 2018-03-09

**Authors:** Jiaxing Ye, Takumi Kobayashi, Masaya Iwata, Hiroshi Tsuda, Masahiro Murakawa

**Affiliations:** 1 National Metrology Institute of Japan (NMIJ), The National Institute of Advanced Industrial Science and Technology (AIST), Tsukuba Central 2, Tsukuba 305-8568, Japan; hiroshi-tsuda@aist.go.jp; 2 Artificial Intelligence Research Center (AIRC), The National Institute of Advanced Industrial Science and Technology (AIST), Tsukuba, Ibaraki 305-8561, Japan; takumi.kobayashi@aist.go.jp (T.K.); m.iwata@aist.go.jp (M.I.); m.murakawa@aist.go.jp (M.M.)

**Keywords:** non-destructive evaluation, hammer sounding, audio signal processing, machine learning, online learning

## Abstract

Developing efficient Artificial Intelligence (AI)-enabled systems to substitute the human role in non-destructive testing is an emerging topic of considerable interest. In this study, we propose a novel hammering response analysis system using online machine learning, which aims at achieving near-human performance in assessment of concrete structures. Current computerized hammer sounding systems commonly employ lab-scale data to validate the models. In practice, however, the response signal patterns can be far more complicated due to varying geometric shapes and materials of structures. To deal with a large variety of unseen data, we propose a sequential treatment for response characterization. More specifically, the proposed system can adaptively update itself to approach human performance in hammering sounding data interpretation. To this end, a two-stage framework has been introduced, including feature extraction and the model updating scheme. Various state-of-the-art online learning algorithms have been reviewed and evaluated for the task. To conduct experimental validation, we collected 10,940 response instances from multiple inspection sites; each sample was annotated by human experts with healthy/defective condition labels. The results demonstrated that the proposed scheme achieved favorable assessment accuracy with high efficiency and low computation load.

## 1. Introduction

Aging infrastructure poses significant challenges to human society. It is indispensable to perform the efficient non-destructive evaluation (NDE) to ensure the safety of those critical structures. In this study, we focus on the hammer sounding test, which is one of the most conventional NDE methods for assessment of concrete structures due to the low-cost and high-efficiency [1,2]. In the setting of hammer sounding, a field engineer generates surface impact using a handy hammer and then determines structural condition by listening to the response. It is evident that such judgement is highly subjective and relies on individual experience; thus, it leaves the assessment judgement results open to human errors. Extensive research efforts were made to develop efficient computerized hammer sounding systems to alleviate human efforts, as well as to eliminate the human errors [3,4,5].

**Figure 1:**
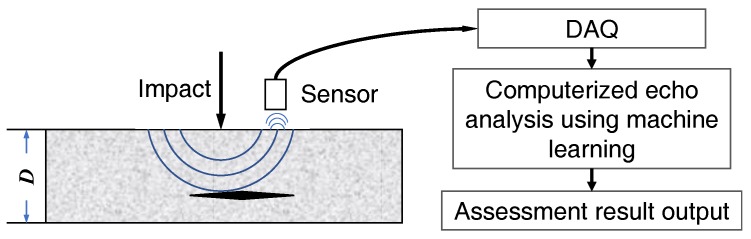
The general processing flow of computerized hammering response investigation.

A typical computerized hammer sounding interpretation system consists of two parts, the hardware and data analysis computing system [6,7]. Impact on the surface produces P- and S-waves that travel into the target structure. Then the responses are captured by a transducer nearby, i.e., an air-coupled microphone. Through data acquisition (DAQ) process, the data is analyzed by a program, and the condition assessment result is presented. Figure 1 shows the schematic flow. This paper mainly addresses the issue of devising an efficient machine learning algorithm for hammer sounding data investigation.

Current learning schemes applied for the hammering response analysis are restricted to the standard batch setting, which assumes that both training and input testing hammer responses reside in the same feature space with the static statistical characteristic; hence, model training can be performed over the pre-collected laboratory-scale database [4,6,7]. In practice, however, such an assumption does not hold. The patterns of response signal can alter significantly with the specifications of concrete structures under evaluation, such as material, shape and years of service [1]. From the viewpoint of machine learning, these factors would make the posterior distribution of the test data drift from that of the pre-collected training samples; thus, degrading the hammer sounding analysis performance.

In this study, we adopt an alternative hypothesis which admits the pre-collected training dataset only covers small range of the complete distribution; moreover, we propose a new formulation of response pattern classification with the online learning paradigm, where efficient model updating schemes have been exploited to minimize the cumulative prediction loss suffered along with the continuous input of data with expert labels. Online learning is a well-established learning scheme which has both theoretical and practical appeals [8,9] and it is particularly well-suited to the hammer sounding, since the large-scale response data can be accessed only in a sequential way.

It is noteworthy that our ultimate goal is to develop an efficient hammering response investigation system with near-human accuracy. In the hammering test, humans are capable of discerning the defect-induced responses of various concrete structures by auditory perception. In this study, we propose an AI-enabled computing system by adopting a formulation of binary classification, which produces labels to indicate healthy or defective concrete, respectively. At validation stage, a loss between the predicted results and expert labels has been employed to compare the performance of the proposed approach to that of humans. The main contributions of the proposed approach can be summarized as follows:

- The objective of this study is to build an efficient hammer sounding analysis system for concrete hammering inspection. To this end, a novel online learning framework had been proposed, which can effectively characterize discriminant information from large-scale response spectrum data in an incremental way.
- Various state-of-the-art online learning algorithms have been reviewed and evaluated for the application of response pattern classification. The side-by-side comparison results can inspire other applications with streaming data input, not limited to the hammer sounding analysis discussed in this study.
- Unlike conventional studies which commonly conduct experiments on laboratory-scale data, a massive dataset has been created during this study, which includes more than 10,000 samples collected from different types of concrete structures. Moreover, each instance has been annotated by professional inspectors with healthy/defective label. The database laid solid fundamentals for learning scheme validation.

## 2. Related Work

In this section, we present a review of the previous studies conducted on hammering response analysis for concrete condition assessment. The review is comprised of two parts: the first is fundamental research towards the impact-echo method, which is closely related to this study, and the latter is recent advancement in developing a computerized hammering response investigation system with machine learning techniques.

### 2.1. Impact-Echo Method and Air-Coupled Hammer Sounding Inspection

The initial literature describing hammering inspection presented is from the 1970s, and subsequently, extensive studies had been carried out in both theoretical and experimental aspects [2,10,11]. Echo signal analysis is commonly performed through Fourier analysis. Although advanced methods have been employed in recent works, such as using Wavelet transform [12] to alleviate poor time-frequency resolution in Fourier spectra and fuse impulse response with 3D laser scanning for delamination detection [13], Fourier analysis still dominates in practice due to its efficient implementation.

**Figure 2:**
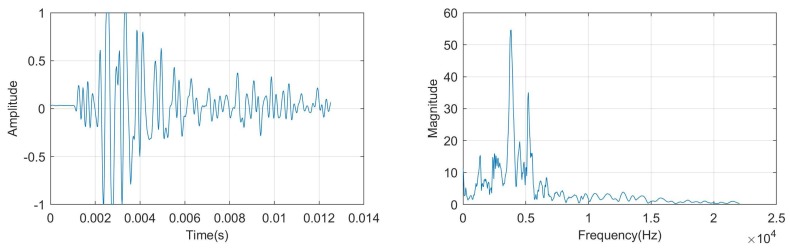
Hammer sounding waveform (**left**) and its Fourier spectrum (**right**).

In Figure 2, we present one example of hammering response waveform and its Fourier spectrum, respectively.

A well-known formula to determine a void beneath surface of concrete is proposed by [1]:

(1) $$d=\beta \frac{C_{p}}{2f_{peak}}$$

where $f_{peak}$ denotes the peak frequency of response signal spectrum, $C_{p}$ is the velocity of the longitudinal, β is constant of 0.96 for plate-shape structures wave according to [1] and *d* represents depth of inside void. However, some recent studies reveal that the availability of formula (1) is constrained by the size and flatness of the defect area, e.g., if void is not parallel to surface, the resonance behaves differently and thus Equation (1) fails to estimate void depth [14]. Impact-echo is initially a contact inspection method, which is quite time-consuming to fix transducer, especially when dealing with large structures. To enhance the efficiency, a new suggestion emerged to apply air-coupled sensor in impact-echo [15,16]. A designated transducer is employed to capture acoustic response from concrete structure through the air. Futhermore, experimental results show the air-coupled sensor is comparable to contact sensors for delamination detection and grouting quality evaluation tasks. Until now, impact-echo method, due to its high diagnosis accuracy and favorable stability, remains to be an active research topic in the non-destructive test field, and efforts will be continuously delivered to the topic.

### 2.2. Data-Driven Hammer Sounding Investigation System for Non-Destructive Test of Concrete Structure

The last five years have seen remarkable progress in machine learning research, and a spreading trend emerged to develop human-level machine learning systems to relieve people from laborious and exhausting tasks in the structural health monitoring field [3,5]. In order to substitute the human role in hammering response interpretation, great efforts were carried out to establish a data-driven machine learning system to discern anomalous responses from healthy ones [4,6,7]. We present a review on current research status as follows.

The early systems commonly tackle the hammer sounding investigation problem with statistical pattern classification, in which the response spectra were used as feature vector and various conventional classifiers were employed, such as Gaussian mixture models (GMM) [4], Artificial Neural Network (ANN) [17] and Support Vector Machines (SVM) [18],to characterize discriminant information of healthy/defective responses. In recent years, significant progress has been made in noise robust feature representation learning. Advanced signal descriptors developed by the bag-of-words model (BoW model) [6] and sparse coding approaches [7] have proved to be effective for anomalous response identification under a hostile acoustic environment. It is noteworthy that this literature commonly assumes that all training and test hammer responses are sampled from the same population; the experimental dataset was confined to be the laboratory-scale as well. It is anticipated to be problematic when we directly apply the analysis model trained by lab-scale data to the practical hammer sounding test, because the pre-collected training data is quite limited to rendering sufficient discriminant information to deal with complex real data.

## 3. The Proposed Online Learning Framework for Hammering Response Pattern Analysis

**Figure 3:**
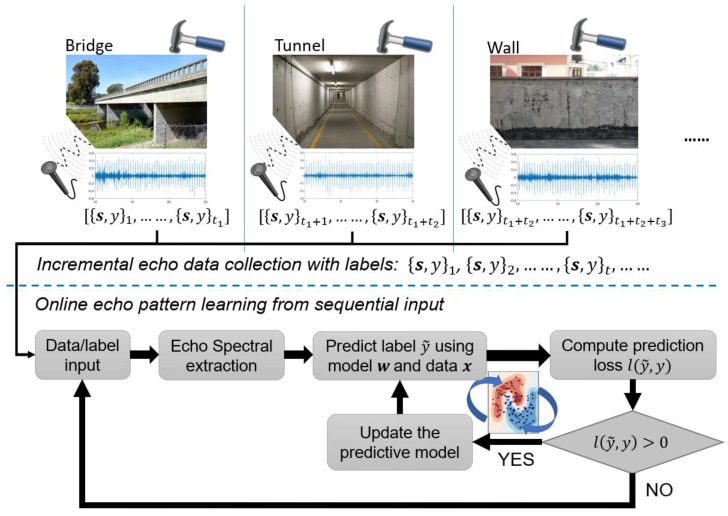
Flow chart of online learning formulation for hammering response investigation.

We introduce the details of the proposed online machine learning-enabled hammering response analysis system in this section. The processing flow has been shown in Figure 3. We assume that the responses have been received in a streaming way ${\left\{s,y\right\}}_{t},t\in \left[1,\ldots ,T\right]$ and all the data had been annotated by a professional inspector with healthy or anomalous labels. Notably, during the data collection, the material, shape, and other specifications of target structures can vary from place to place. For instance, in Figure 3 the inspection place shifted from bridge to tunnel, meanwhile the captured data/label indexes range varied from $1∼t_{1}$ to $t_{1}+1∼t_{1}+t_{2}$, respectively. Our goal is to continuously update the analysis model so as to achieve near-human performance for flaw-induced hammering response identification. To this end, it is crucial to devise the efficient online learning scheme and various state-of-the-art algorithms have been reviewed and compared. The details are presented as follows.

### 3.1. Feature Extraction

**Figure 4:**
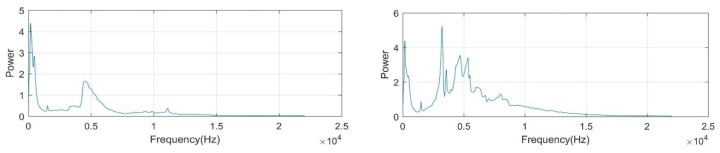
Examples of hammering response spectrum: normal (**left**) and defective cases (**right**).

Over decades, spectral analysis has been the dominant method for hammering response characterization [10,11], in which Fourier transform (FT) is employed to generate the spectrum. Then, further pattern investigation can be performed. The Fourier analysis can be expressed as:

(2) $$x\left(f\right)=\int_{-\inf}^{\inf}s\left(t\right)e^{-2\pi ift}dt$$

where s(t) is waveform of hammering response to be analyzed and x(f) is the extracted spectrum. In the following contents, we will use $x_{t}\in R^{d},t\in \left[1,\ldots ,T\right]$ to denote the spectrum collected at time stamp *t*, with *d* frequency-bins. Our pattern classification process is based on Fourier spectrum representation of response signal. In Figure 4, we present two spectrum examples, which were collected from normal and defective concrete structures, respectively. According to the plots, differences in spectral distributions can be clearly observed. In addition, at a low frequency region below 500 Hz, high noise power can be seen which is irrelevant to the specimen. We employed high pass filter to eliminate the ambient noises presented in the band lower than 500 Hz. Meanwhile, to reduce feature dimension, we also discarded the frequency bands higher than 15 kHz, since there existed no discriminant information.

### 3.2. Online Learning Algorithms in Evaluation

As the core feature of this research, online learning algorithms are employed to deal with the case that hammering response data (with expert annotations) arrives incrementally with time stamps. Concretely, at time *t* , the online learning algorithm analyses the input data and expert label, i.e., $\left\{{x,y}_{t}\right\}$, through three steps: the first is to predict its label ${\hat{y}}_{t}\in \left\{-1,+1\right\}$, in which the two digits represent defective and healthy status, respectively. Then, we compare the predicted label ${\hat{y}}_{t}$ with true label $y_{t}\in \left\{-1,+1\right\}$ by using a well-defined loss function $l(y_{t},{\hat{y}}_{t})$. Finally, if the computed prediction loss exceeds a threshold, the classification model will be updated in an analytical way. Overall, the cumulative mistake through whole data stream can be minimized. In this section, we first present a general algorithmic framework of online machine learning for hammer sounding discriminant analysis in Algorithm 1. Then, we explicitly introduce the algorithms employed for this application.

**Algorithrm 1.** Online Learning ($x_{t},y_{t},w_{t}$)**Initialization**
**w**_1_ ← 0 **for**
*t* = 1, 2, …, *T*  $\mathrm{do}\{\begin{array}{l} \text{input}\;\text{hammering}\;\text{response}\;\text{is}\;\text{received}\;\text{with}\;\text{label}:\;x_{t}\in X,\;y_{t}\in Y\\ \text{predict}\;\text{label}\;\text{of}\;\text{input}\;\text{hammering}\;\text{response}:\;{\hat{y}}_{t}=\text{sign}(f(x_{t};\;w_{t}))\\ \text{compute}\;\text{the}\;\text{prediction}\;\text{loss}:\;l(w_{t};\;(x_{t};\;w_{t}))\\ \mathrm{if}\;l(w_{t};\;(x_{t};\;w_{t}))>0\\ \;\mathrm{then}\;\text{update}\;\text{the}\;\text{classification}\;\text{model}:\\ w_{t+1}\leftarrow \Delta ((w_{t};\;(x_{t};\;w_{t}))) \end{array}$ **return** (**w**_*t* + 1_)

#### 3.2.1. Perceptron

The perception algorithm is the initial method for online learning [19]. Given the linearly separable data, the method can converge to a hyperplane to shatter the different classes in a finite number of updates. The prediction function of perception is very simple: ${\hat{y}}_{t}=\mathrm{sign}\left(w^{⊺}x_{t}\right)$ and the updating rule will be conducted as follows:

(3) $$w_{t+1}=w+y_{t}x_{t}\;\mathrm{if}\;{\hat{y}}_{t}\neq y_{t}$$

There is neither parameter nor optimization constraints in the perception algorithm. The perceptron algorithm has several limitations. First, it can only classify linearly separable sets of vectors. If the class-conditional data distribution is inherently nonlinear, perceptron will never reach a point where all vectors are classified properly. Second, since there is no constraint applied during model training, perceptron is vulnerable to noise. To alleviate the problems, substantial modification had been carried out. Representative works can be referred in [9].

#### 3.2.2. Online Gradient Descent (OGD)

Gradient descent updating is another efficient approach for online learning [20]. In this evaluation, we selected logistic loss to measure the prediction error:

(4) $$l\left(w;\left(w_{t},y_{t}\right)\right)=\log\left(1+\exp\left(-y_{t}\left(w_{t}{\cdot x}_{t}\right)\right)\right)$$

Subsequently, the updating rule can be represented as:

(5) $$\begin{array}{c} w_{t+1}=w_{t}+\eta_{t}y_{t}x_{t}\cdot \frac{1}{1+\exp(y_{t}\left(w_{t}\cdot x_{t}\right))},\eta_{t}=C/\sqrt{t} \end{array}$$

#### 3.2.3. Passive-Aggressive Learning Algorithm [PA]

Passive-Aggressive Learning Algorithm [PA] is one state-of-the-art first order online learning approach. The optimization formulation can be expressed as follows:

(6) $$w_{t+1}=\underset{w\in R^{d}}{\mathrm{argmin}}\frac{1}{2}||w-w_{t}{\left|\right|}^{2},\;s.t.l\left(w;\left(w_{t},y_{t}\right)\right)$$

where the loss function is based on the hinge loss:

(7) $$l\left(w;\left(w_{t},y_{t}\right)\right)=\left\{\begin{array}{cc} 0 & \mathrm{if}\;y_{t}\left({w\cdot x}_{t}\right)\geq 1\\ 1-y_{t}\left(w_{t},y_{t}\right) & \mathrm{otherwise} \end{array}\right.$$

The updating rule can be derived analytically:

(8) $$w_{t+1}=w_{t}+\eta_{t}^{PA}y_{t}x_{t},\;\mathrm{where}\;\eta_{t}^{PA}=\frac{l(w_{t};\left(w_{t},y_{t}\right)))}{\left|\right|w_{t}{\left|\right|}^{2}}.$$

In addition, several variants of PA method had been investigated [21]. The core idea is to add slack variable ξ-induced penalty to handle non-separable cases.

#### 3.2.4. The Second Order Perception (SOP)

Aiming at better characterizing the hammering response structure, advanced second-order online learning approaches were developed. Unlike the above-mentioned first-order algorithms, the second-order online learning is designated to exploit the underlying relationship between features. Concretely, it assumes the weight vector exhibits Gaussian distribution w∼N(μ,Σ). At initialization stage, two additional hyperparameters are commonly set to $w_{1}=0,\Sigma_{1}=aI$. Furthermore, the prediction function is noted as:

(9) $${\hat{y}}_{t}=\mathrm{sign}\left(w^{⊺}x_{t}\right),\;w_{t}={\left(\Sigma_{t}+x_{t}x_{t}^{⊺}\right)}^{-1}\mu_{t}$$

The following updating process is conducted as the predicted label is inconsistent with the true label:

(10) $$\mu_{t+1}=\mu_{t}+y_{t}x_{t},\;\Sigma_{t+1}=\Sigma_{t}+x_{t}x_{t}^{⊺}$$

A representative work for second order perception can be referred to [22].

#### 3.2.5. The Confidence-Weighted Learning Algorithm (CW)

CW method is an advanced second-order online learning [23]. In contrast to SOP approach, CW methods perform the Kullback-Leibler divergence minimization between the new weight distribution and the old one with constraint so that the probability of correct classification can be improved. The updating rule of CW is shown as below:

(11) $$\left(\mu_{t+1},\Sigma_{t+1}\right)=\underset{\mu ,\Sigma }{\mathrm{argmin}}D_{KL}\left(N\left(\mu ,\Sigma \right),N\left(\mu_{t},\Sigma_{t}\right)\right),s.t.Pr_{w∼N(\mu ,\Sigma )}\left[y_{t}\left({w\cdot x}_{t}\right)\right]\geq \eta \;$$

A closed-form solution can be derived as: $\mu_{t+1}=\mu_{t}+\alpha_{t}y_{t}\Sigma_{t}x_{t}$; $\Sigma_{t+1}=\Sigma_{t}-\beta_{t}\Sigma_{t}x_{t}^{⊺}x_{t}\Sigma_{t}$, where the updating coefficients can be calculated as follows: $\alpha_{t}=\max\left\{0,\frac{1}{v_{t}\zeta }\left(-m_{t}\psi +\sqrt{m_{t}^{2}\frac{\phi^{4}}{4}+v_{t}\phi^{2}\zeta }\right)\right\}$; $\beta_{t}=\alpha_{t}\phi /\left(\sqrt{u_{t}}+v_{t}\alpha_{t}\phi \right)$. More detail parameters setting discussion can be found in [23].

#### 3.2.6. Adaptive Regularization of Weight Vectors (AROW)

Regularization is regarded as a useful trick to enhance both accuracy and robustness of the online learning algorithm. AROW method added an adaptive regulizer to restrict the sudden changes of weight during online learning [24]. The formulation of AROW is presented as follows:

(12) $$\left(\mu_{t+1},\Sigma_{t+1}\right)=\underset{\mu ,\Sigma }{\mathrm{argmin}}D_{KL}\left(N\left(\mu ,\Sigma \right),N\left(\mu_{t},\Sigma_{t}\right)\right)+\frac{1}{2\gamma }l^{2}\left(\mu ;\left(x_{t},y_{t}\right)\right)+\frac{1}{2\gamma }x_{t}^{⊺}\Sigma xt$$

where

(13) $$l^{2}(\mu ;\left(x_{t},y_{t}\right)={\left(\max\left\{0,1-y_{t}\left(\mu \cdot x_{t}\right)\right\}\right)}^{2}$$

The updating coefficients can be obtained by solving optimization problem:

(14) $$\mu_{t+1}=\mu_{t}+\alpha_{t}\Sigma_{t}y_{t}x_{t},\;\Sigma_{t+1}=\Sigma_{t}-\beta_{t}\Sigma_{t}x_{t}x_{t}^{⊺}\Sigma_{t}$$

(15) $$\alpha_{t}=l\left(\mu_{t};\left(x_{t},y_{t}\right)\right)\beta_{t},\;\beta_{t}=\frac{1}{x_{t}^{⊺}\Sigma_{t}x_{t}+r}$$

#### 3.2.7. Soft Confidence-Weighted Learning (SCW-II)

SCW is a more advanced second-order learning algorithm that improves over the original CW by adding the capability to handles the non-separable cases, and also improves over AROW by adding the adaptive margin property [25]. The classification suffer loss for input hammer sounding data is defined as $l_{t}=\max\left\{0,1-y_{t}w_{t}^{⊺}x_{t}\right\}$. If $l_{t}>0$, the classification model will be updated:

(16) $$\mu_{t+1}=\mu_{t}+\alpha_{t}y_{t}\Sigma_{t}x_{t};\;\Sigma_{t+1}=\Sigma_{t}-\beta_{t}\Sigma_{t}x_{t}^{⊺}x_{t}\Sigma_{t},$$

where

(17) $$\begin{array}{c} \alpha_{t}=\min\left\{C,\max\left\{0,\frac{1}{v_{t}\zeta }\left(-m_{t}\psi +\sqrt{m_{t}^{2}\frac{\phi^{4}}{4}+v_{t}\phi^{2}\zeta }\right)\right\}\right\};\;\beta_{t}=\alpha_{t}\phi /\left(\sqrt{u_{t}}+v_{t}\alpha_{t}\phi \right)\\ u_{t}=\frac{1}{4}{\left(-v_{t}\alpha_{t}\phi +\sqrt{\alpha_{t}^{2}v_{t}^{2}\phi^{2}+4v_{t}}\right)}^{2},\;,v_{t}=x_{t}^{⊺}\Sigma_{t}x_{t},m_{t}=y_{t}\left(\mu_{t}\cdot x_{t}\right)\\ \gamma_{t}=\phi \sqrt{\phi^{2}m_{t}^{2}v_{t}^{2}+4n_{t}v_{t}\left(n_{t}+v_{t}\phi^{2}\right)}\;\mathrm{and}\;n_{t}=v_{t}+\frac{1}{2C} \end{array}$$

### 3.3. Hammering Response Data Visualization

Data visualization is widely recognized as one integral part of today’s data analysis systems, which makes complex data more accessible, understandable and usable. In our hammer sounding pattern investigation system, we incorporate data visualization function so as to let end-users browse and understand the massive data distributions. We adopted the fundamental method principal component analysis (PCA), which is a standard way of visualizing data. The basic principle of PCA is to find the low dimension linear subspace such that the variations of data can be maximized. The detail procedures can be found in [26]. We present the whole hammering response data visualization results in the experimental analysis section.

## 4. Experimental Validations

### 4.1. Data Collection

**Figure 5:**
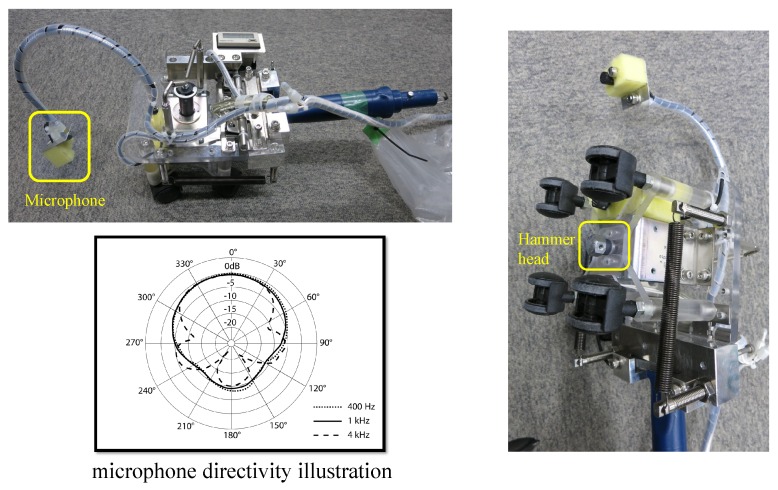
The hammer device and microphone used for data capture (**upper**, **right**) and the microphone directivity illustration.

**Figure 6:**
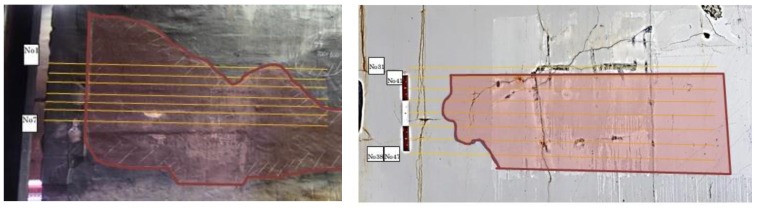
Photos of two working sites for hammering response data capture.

In this section, we introduce the hammer sounding dataset we created to evaluate the proposed system. First, we present the hardware we used for data collection in Figure 5, including solenoid hammer device and microphone. Then, Table 1 shows more detail specifications. For response data recording, the sampling rate was set to 44.1 kHz and resolution was fixed to 16-bit depth. We visited 12 inspection sites to capture hammer sounding data. Meanwhile, binary expert annotations, i.e., the response indicating normal or anomalous concrete, has also been collected. In Figure 6, we show the photos of two inspection sites. The defective area had been tagged with pink color by inspector. In addition, we marked multiple parallel lines in yellow, which explains the trace of hammering. Scanning speed was around 80 centimeters per minute (cm/min). The hammer area varies with location. As a result, we obtained 10,940 annotated hammer responses, among which 9349 are normal and 1591 are anomalous instances, respectively. The dataset laid fundamentals for further numerical analysis.

### 4.2. Experimental Settings

At hammer response feature extraction stage, we determine the Fourier analysis window length to be 1024. Band pass filter is applied to focus on the frequencies ranging from 500 to 15,000 Hz. At online learning stage, parameter tuning plays a key role in achieving accurate pattern classification. In this study, we evaluate seven state-of-the-art online learning approaches with the massive data. The detail parameter settings are presented in Table 2.

The first parameter *C* governs the trade-off between the fitting loss term and regularization term in machine learning model training. In the second order algorithms, the parameter α=1 is used to initialize the covariance matrix, i.e., Σ=α∗I, where *I* is identity matrix. Parameter η is used to define loss function in the confidence-weighted learning algorithms, i.e., in CW and SCW-II. The experiments had been performed in the same vein as a real scenario, in which the labeled data was fed to the online learning system in a sequential manner. The experiments were conducted over 20 random permutations for the whole dataset. At each iteration, we divided the dataset into 15 sub data sets. During online learning, we recorded the evaluation results, i.e., response-wise classification accuracies and computation time costs, when one subset had been processed. Those information will help us understand the learning behavior of the algorithm. Finally, the results are presented by averaging of total 20 trials.

### 4.3. Echo Data Visualization

**Figure 7:**
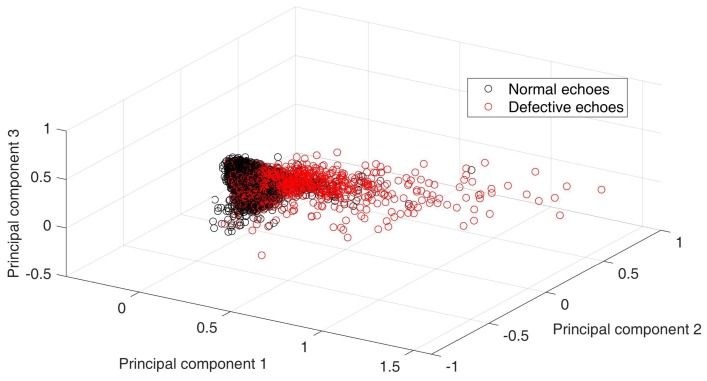
Visualization of hammering response dataset using principal component analysis (PCA).

As introduced in Section 3.3, data visualization is a useful approach to understanding the data. In Figure 7, we present the distribution of the dataset using principal component analysis (PCA). In the visualization, binary class labels were noted with different colors, i.e., the normal hammer responses were marked with black and flaw-induced ones were colored in red. According to the distribution plot, we have several major findings: 1. damage-induced responses produced more scattered distribution compared to that of healthy ones. It is reasonable because the damaged concrete usually generated more complex spectrum; 2. The boundary between normal and anomalous responses is not clear; in other words, there exists strong non-linearity between the two-class distribution. From the machine learning aspect, the methods which were designated to deal with the inherently nonlinear data may perform superiorly. Grounded on the above understanding of the data collection, we start the algorithmic analysis as follows.

### 4.4. Empirical Evaluation Results

**Figure 8:**
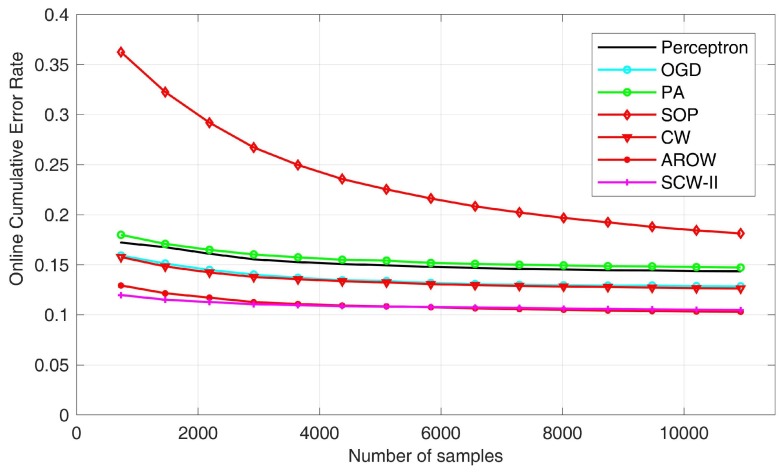
Summary of online cumulative classification error rate.

In this part, we present results of experimental validation. The comparison has been drawn of three aspects: normal/defective hammer response classification accuracy, processing efficiency and computation complexity. As for the first comparison—accuracy, we adopted two metrics: mistake rate transition curve and the cumulative classification error rate. It can be anticipated that with more data being examined, the mistake rate would decrease monotonically. To exploit the performance of online analysis models, we presented the cumulative error rate after the whole online learning process was done. Figure 8 exhibited the overall errors statistics during the online learning process. First of all, by examining the overall mistakes, we found that second-order algorithms, i.e., SOP, CW, AROW and SCW-II usually outperform first-order algorithms, including perception, OGD and PA; also margin based algorithms, such as CW and SCW-II, usually outperform non-margin based methods.

**Figure 9:**
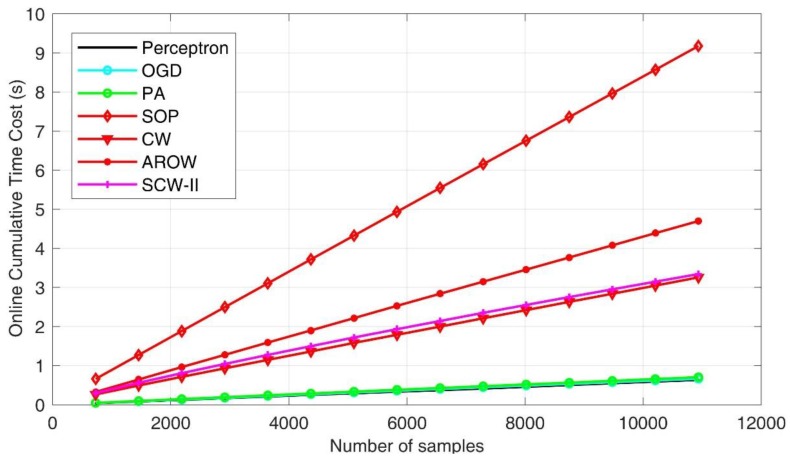
Processing time cost comparison.

**Figure 10:**
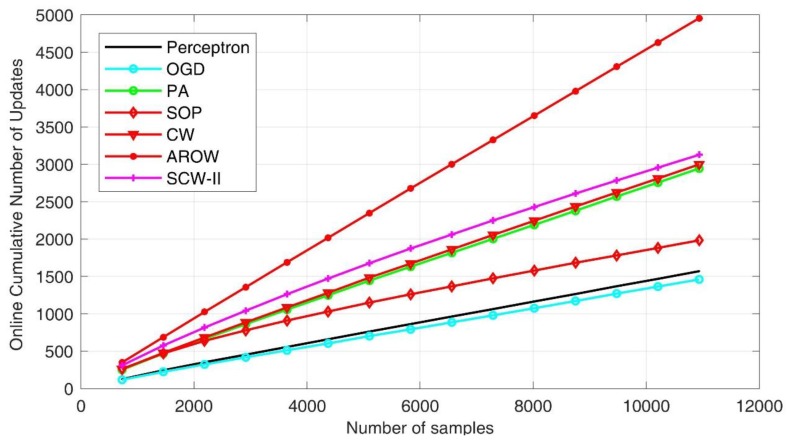
Comparison of number of updating steps.

As for the second evaluation criteria—computation efficiency, we presented the cumulative time costs for all the seven online learning algorithms under evaluation in Figure 9. We found that the first-order schemes exhibited superior efficiency due to their simpler formulation. The SOP method took the longest time in process, which is one of initial second order approaches. Confident-weighted learning methods, including both CW and SCW, achieved favorable performance in balancing accuracy and efficiency. To demonstrate the complexity of online learning algorithms, we further showed cumulative number of updates. In general, fewer update steps indicates the algorithm is efficient in establishing a more robust pattern classification hyperplane such that input feature distribution shift can be accommodated. By examining Figure 10, we can see that first order methods usually produced smaller numbers of updates. However, the classification accuracies were inferior. AROW scheme made significantly larger number of updates, which can induce high time cost in processing.

To clarify the comparison, we further prepared Table 3 to summarize the key experimental results, including cumulative mistake rate, size of support vectors (SVs) and cpu run time. Such quantitative information is complementary to the above charts. From the table, we found that SCW-II outperformed all other methods in hammer sounding pattern classification accuracy. Meanwhile, the method achieved superior efficiency among all the second order online learning algorithms. Besides, we also investigated the number of support vectors (SVs) used by different learning schemes. SVs are defined as the samples used to determined max-margin hyperplane for classification. Since the hammer responses are highly non-linear, we can see that larger numbers of SVs had been used at classification stage.

As a result, among all the compared algorithms, SCW-II produced the best performance in terms of accuracy; for other metrics including number of updates, and running time cost, it also outperformed other second order methods. Therefore, the method can be the optimal selection for the application of hammering response investigation. It is noteworthy that errors may exist in the labels, because inspectors usually take location information into account. It can be regarded as performing region-based smoothing over each individual expert label nearby. In contrast, our quantitative evaluation was conducted in a point-wise way. Such a factor can be one major reason that led to the error rate over 10%. The empirical evaluation validated the effectiveness of the online learning approach.

## 5. Discussion and Conclusions

To tackle the growing problem of aging infrastructure, the need for non-destructive evaluation (NDE) methods with high efficiency and low-cost has become a priority. In this context, engineers are still using these old-fashioned hammer sounding tests that are very subjective and open to human error. In this paper, we attempted to develop the efficient learning framework which is able to mimic human expert ability in hammering response investigation. Specifically, we formulate the task by using binary statistical classification between flaw-induced responses and normal ones. In order to deal with large-scale data, we employed online learning algorithm which can adaptively update itself so as to minimize the cumulative error rate as data and label are received in a sequential manner. To validate the proposed system, we created a massive dataset with professional annotations. The experimental result demonstrated the effectiveness of the proposed learning approach.

Moreover, the proposed system presented several favourable characteristics to facilitate the practical inspection: 1. An objective criterion can be established by using advanced machine learning algorithms for hammer response interpretation, which can effectively eliminate man-made errors; 2. Compared with human inspection, the proposed system exhibited higher efficiency in processing and it can be performed in real time; 3. The online learning framework can be adapted to deal with other types of impact-echo data, not limited to the hammer sounding application addressed in this study. In the next stage, we hope to upgrade the hardware part, such as employing wireless/wearable MIC; also, it can be anticipated that as we collect more hammering sounding data, the condition assessment precision will be further improved. 

## Figures and Tables

**Table 1:** Summary of the parameter setting by algorithms.

	Device	Specification
1	Hammer device	Solenoid: Takaha Kikou Co., Ltd., CB15670033
2	Transducer	Low-cost condenser microphone: ECM PC60
3	Recorder	Olympus Voice-Trek V-803

**Table 2:** Summary of the parameter setting by algorithms.

Index	Algorithm	C	α	η	Others
1	Perceptron	/	/	/	parameter free
2	OGD	C=1	/	/	$\eta_{t}=C/\sqrt{t}$
3	PA	/	/	/	parameter free
4	SOP	/	α=1	/	parameter free
5	CW	/	α=1	η=0.7	Σ=a∗I
6	AROW	C=1	α=1	/	r=C,Σ=α∗I
7	SCW-II	C=1	α=1	η=0.75	Σ=a∗I

**Table 3:** Summary of all experimental results.

Algorithm:	Mistake Rate (M ± Std)	Size of SVs (M ± Std)	Cpu Time (M ± Std)
Perceptron	0.144 ± 0.002	1570.8 ± 25.2	0.647 ± 0.056
OGD	0.128 ± 0.005	1460.7 ± 59.3	0.718 ± 0.045
PA	0.147 ± 0.002	2945.8 ± 41.2	0.699 ± 0.037
SOP	0.181 ± 0.002	1983.8 ± 27.0	9.708 ± 0.608
CW	0.126 ± 0.002	3000.1 ± 36.6	3.460 ± 0.248
AROW	0.115 ± 0.004	6378.9 ± 278.2	6.357 ± 0.429
SCW-II	0.105 ± 0.002	3128.4 ± 61.6	3.577 ± 0.244
